# Peer Review and Manuscript Management in Scientific Journals: Guidelines for Good Practice

**DOI:** 10.3352/jeehp.2008.5.5

**Published:** 2008-12-22

**Authors:** Sun Huh

**Affiliations:** Department of Parasitology, College of Medicine and Institute of Medial Education, Hallym University, Chuncheon, Korea.

Ms. Irene Hames had served as managing editor of *The Plant Journal* for 18 yr. Her career tells us that she is a professional managing editor. I discovered this book when I was preparing The Editor's Academy, a three-day intensive training course for medical editors in Korea held in February 2008. The content of the book was a great source for the workshop. Furthermore, peer review, online submission, and misconduct in scientific research and publishing are interesting topics that recently has been of concerned to editors. The simple, lucid organization of this book includes the description of golden rules in the text and a summary in the appendix. This book consists of nine chapters: Introduction; Peer Review Process-How to Get Going; Manuscript Submission and Initial Check on Completeness and Suitability; Full Review Process; Decision Making Process for Reviewed Manuscripts; Moving to Online Submission and Review; Reviewers-A Precious Resource; Obligations and Responsibilities of the People Involved in Peer Review; and Misconduct in Scientific and Publishing-What it is and How to Deal with it.

In the introductory chapter, the author explained two factors that led to the spread of peer review. First, submissions to scientific journals are burgeoning. Second, editors are no longer experts in all areas of their specialties. The increase in submissions likely originated from increases in research funds and the use of publications to evaluate members of research institutes worldwide. The first page of the book includes a box highlighting the definition of peer review by the International Committee of Medical Editors: *"Critical assessment of manuscripts submitted to journals by experts who are not part of the editorial staff."* On the third page, the first golden rule appears: *"Editors are responsible for ensuring the quality of their journals and that what is reported is ethical, accurate, and relevant to their readership."* Throughout the book, boxes, bewares, and golden rules are frequently given to reinforce the messages of the text. The writing is fluent and warm, like a conversation with the reader, and is another reason why this book is strongly recommended to novice editors or editorial board members.

The content of chapters two to five concerns the review process and decision making, and the remaining chapters deal with online submission, routine work required of authors, and the reviewers' work. Chapter two presents a well-drawn flow chart of the submission and review process on page 10. In addition, the organization of the editorial office and the title and role of each member of the office are described (pages 12-13). Although not all editors/publishers can recruit an extensive office staff, especially when the journal is published by a non-profit scientific society, these titles and roles should be kept. If sufficient finances are available, full- or part-time editorial assistants should be considered to improve the editorial quality. In Korea, medical editors are usually very active clinicians or researchers who would quickly become exhausted without help from editorial assistants. Chapter four includes a checklist of the full review process (page 62). Ten items are explained to help the novice reviewer deal with a manuscript, such as scope, study objective, study design, and soundness of the results. Editors sometimes evaluate the quality of their reviewers, as is the case with the 'Review Quality Instruments' used by the British Medical Journal. Pages 80-81 Box 4.3 presents advice to the reviewer on how to prepare an ideal report for the authors and provides lucid guidelines for reviewers. In Chapter 5, Table 5.2 summarizes decision making after a review, including reviewer recommendations such as acceptable as stands, acceptable with minor revision, acceptable with major revision, reject with resubmission invited, and reject with no encouragement of resubmission (page 95). Another editorial task is to deal with authors' appeals. An editor should not be surprised when an author appeals, and automatic dismissal of appeals is not recommended. The editor should listen to the author and examine whether the reviewer made a mistake. Perhaps another reviewer can review the manuscript, or the editor can conclude that the manuscript is sound but explain how it falls outside the journal's scope.

Chapter 6 addresses online submission in detail. Box 6.1 contains a useful checklist for selecting an online submission system (page 121). After implementing an online submission system, the number of submissions usually increases by around 25%, including an increased number of low-quality manuscripts, so it is important that the consistency of the review process is ensured.

The obligations and responsibilities of reviewers and editors are discussed in Chapter 8. One of the most important issues is conflicts of interest. Golden Rule 12 describes the principle lucidly: *"No conflict of interest or prejudice must be allowed to influence the submission of a manuscript, its review, or the decision on whether it should be published"* (page 164). The existence of a conflict of interest does not mean that the manuscript is wrong or biased. The key is the disclosure of conflict of interest, so that the information can be used in the review process. This process increases the transparency of the peer-review process.

The last chapter deals with misconduct in research and publication. This is still an ongoing issue, not only in Korea, but in scholarly society worldwide. Some examples of fabrication and falsification are presented, such as the cases of Jan Hendrik Schon, who fabricated or falsified data in at least 16 papers on the physical sciences, and Hwang Woo Suk, whose claims, published in *Science*, to have cloned human embryos and harvested stem cells from them were found to be fraudulent. Plagiarism, duplicate publication, multiple submissions, digital image manipulation, authorship irregularities, and other issues are discussed. In addition, reviewer misconduct is addressed, such as failure to disclose a conflict of interest, disclosing confidential information, usurping authors' ideas, delaying reviews, or damaging the reputation of authors.

Four appendices help to recall the full text: Golden Rules and the Peer Review Good-practice Checklist; Examples of Checklists, Guidance for Reviewers and Editorial Letters; Useful Websites; and Alternative Methods of Peer Review. All of this well-arranged information provides useful guidelines for practical editorial work. Many forms used by several famous journals are presented as a reference for editors.

The text of this book includes material that editors deal with every day, and it helps to remind me of the editorial process. Editors should also be trained as researchers. Unfortunately, there is no well-organized training course in Korea. Only the Korean Association of Medical Journal Editors provides courses for editors. This wonderful work by Ms. Hames can be used as a textbook in courses for both experienced and novice editors, and I trust that it is what Ms. Hames intended when she prepared this beautiful book. Every scientific editor should read it.

